# Comparing the effects of pulsed and radiofrequency catheter ablation on quality of life, anxiety, and depression of patients with paroxysmal supraventricular tachycardia: a single-center, randomized, single-blind, standard-controlled trial

**DOI:** 10.1186/s13063-024-07971-8

**Published:** 2024-02-24

**Authors:** Ying Du, Shanshan Ma, Pan Yue, Ying Xu, Ya Wen, Mingzhu Ji, Lingxiao He, Dengbin Liao

**Affiliations:** 1 Department of Orthopedic Surgery, West China Hospital, Sichuan University/West China School of Nursing, Sichuan University, Chengdu, China; 2 Trauma Center, West China Hospital, Sichuan University/West China School of Nursing, Sichuan University, Chengdu, China; 3 Department of Cardiology, West China Hospital, Sichuan University/West China School of Nursing, Sichuan University, Chengdu, China; 4 Department of Pharmacy, Chengdu University of Traditional Chinese Medicine, Chengdu, China

**Keywords:** Paroxysmal supraventricular tachycardia, Pulsed field ablation, Radiofrequency catheter ablation, Quality of life, Anxiety, Depression

## Abstract

**Background:**

Radiofrequency catheter ablation (RFCA) may lead to decreased quality of life (QOL) and increased anxiety and depression in patients with paroxysmal supraventricular tachycardia (PSVT), possibly due to the lack of selectivity of the ablation tissue and the long ablation time. In recent years, pulsed field ablation (PFA) has been used for the first time in China to treat PSVT patients because of its ability to ablate abnormal tissue sites in a precise and transient manner. This study was conducted to compare the effects of PFA and RFCA on QOL and psychological symptoms of PSVT patients.

**Methods:**

We have designed a single-center, randomized, single-blind, standard-controlled trial. A total of 50 participants who met the eligibility criteria would be randomly allocated into the PFA group or RFCA group in a 1:1 ratio. All participants were assessed using the 36-Item Short-Form Health Survey (SF-36) and the Hospital Anxiety and Depression Scale (HADS) at pre-procedure (T0), post-procedure (T1), and 3 months post-procedure (T2). The SPSS 21.0 software was used to analyze the data through Wilcoxon and Fisher’s exact tests and repeated measures ANOVA.

**Results:**

Twenty-five in the PFA group and 24 in the RFCA group completed the trial. SF-36: (1) Between-group comparison: At T1, PFA group had significantly higher SF-36 scores on physiological function (PF) and general health (GH) than RFCA group, with a treatment difference of 5.61 points and 18.51 points(*P* < 0.05). (2) Within-group comparison: We found that in the PFA and RFCA groups, T2 showed significant improvement in the remaining 6 subscales of the SF-36 scale compared to T1 and T0 (*P* < 0.05), except for body pain (BP) and social function (SF) scores. HADS: (1) Between-group comparison: no significant difference (*P* > 0.05). (2) Within-group comparison: The HADS scores of the PFA and RFCA groups were statistically significant at T2 compared to T0 and T1 (*P* < 0.05).

**Conclusions:**

Our study provided new and meaningful evidence that PFA was effective in significantly improving QOL and decreasing anxiety and depression in PFA patients.

**Trial registration:**

Chinese Clinical Trial Registry, ChiCTR2200060272.

**Supplementary Information:**

The online version contains supplementary material available at 10.1186/s13063-024-07971-8.

## Background

Paroxysmal supraventricular tachycardia (PSVT) is a paroxysmal rapid and regular ectopic rhythm, mostly originating from tachycardia in the atrial or atrioventricular junction, mostly due to a reentrant mechanism [1]. According to the Marshfield Epidemiological Survey in Wisconsin, USA, the annual incidence of PSVT could reach 35/100,000, with a prevalence of approximately 2.5/1000 [2]. PSVT patients often complain of palpitations, chest discomfort, polyuria, and sweating, which affect the quality of life (QOL) of patients [3, 4]. A growing number of studies have shown that the symptoms and complications of PSVT cause decreased QOL and psychosocial problems that cannot be ignored [3]. The treatment of PSVT is not only about improving clinical symptoms but also about improving prognosis, QOL, and psychological symptoms.

In 1987, radiofrequency catheter ablation (RFCA) was first successfully applied in adults for the treatment of atrioventricular reentrant tachycardia [5]. RFCA is performed by puncturing the femoral vein to deliver the catheter into the heart, finding the anomalous site through a mapping technique, and releasing energy through the catheter tip to eliminate the anomalous site, thus blocking the anomalous pathway for therapeutic purposes [6]. However, RFCA lacks selectivity in the destruction of tissue in the ablation zone and may cause damage to adjacent tissues such as the esophagus and phrenic nerve, resulting in a reduced QOL for the patient [7]. Meanwhile, the intracardiac electrophysiological examination may be painful for most patients, who often has psychological reactions such as nervousness and anxiety [8]. Adverse psychological symptoms may lead to prolonged QT intervals, ventricular arrhythmias and even sudden death [9, 10].

In recent years, pulsed field ablation (PFA) has developed rapidly, using high-voltage electrical pulsed fields to act on tissue within the heart chambers, forming irreversible electroporation and causing myocardial cell death to eliminate and prevent abnormal sites [11]. PFA is a non-thermal ablation method that selectively damages cardiomyocytes and does not directly touch the ablated tissue during the ablation process, so PFA does not damage the ablated surrounding tissue and is now widely used in the field of atrial fibrillation [12]. A proof-of-concept study using a 12-Fr deflectable PFA catheter and a deflectable sheath guided by electroanatomic mapping to ablate the right and left ventricles of four healthy pigs found that PFA resulted in a significant reduction in electrograms without ventricular arrhythmias [13]. Another animal study demonstrated that PFA could rapidly, safely, and effectively ablate abnormal cardiac myocytes surviving in the left ventricular substrate of 10 swine with left ventricular myocardial infarction [14]. In addition, the authors concluded that PFA was promising for the treatment of infarct-related ventricular tachycardia in humans. A study compared the effects of PFA and RFCA on the esophagus of pigs and found that PFA did not damage the esophagus, whereas RFCA caused a range of esophageal lesions, including esophageal fistulas, ulcers, and abscesses [15]. These animal experiments indicated that PFA was safe and feasible for cardiac ablation therapy. However, the application of PFA in PSVT patients is still in the exploratory stage.

It has been shown that RFCA could improve QOL and adverse psychological symptoms of patients with PSVT. A prospective study [16] found that PSVT patients treated with RFCA had a significant improvement in QOL and a reduction in patients’ physical and emotional perceived limitations at 1 year. As demonstrated in two previous studies [17, 18], RFCA improved the QOL of patients with PSVT and had good lifetime cost-effectiveness. However, even though PFA was less damaging to patients’ tissues compared to RFCA [19], little attention has been paid to the effects of PFA on PSVT-related QOL and psychological symptoms in various studies.

In 2022, we successfully used the PFA technology to treat PSVT, and the results confirmed that PFA was feasible and safe [20]. In the current single-center, randomized, single-blind, standardized comparative trial, we aimed to compare the effects of 2 treatment strategies (PFA and RFCA) on QOL and psychological symptoms with PSVT patients. We hypothesized that among a broad range of participants who were symptomatic and elected surgical treatment, patients treated with PFA would demonstrate favorable QOL and psychological outcomes. The primary outcome was QOL, and the secondary outcome was psychological symptoms. Our findings possibly provide a reference for the application of PFA in adults with PSVT and facilitate the development of new technologies.

## Methods

### Design and participants

This was a single-center, randomized, single-blind, standard-controlled study of adult PSVT patients recruited from the Department of Cardiology, West China Hospital, Sichuan University, between April 2022 and March 2023. The study protocol was approved by the ethics committee of West China Hospital, Sichuan University (No. 2022-766) and complied with the ethical guidelines of the Declaration of Helsinki. At the same time, we used CONSORT randomized controlled trials reporting guidelines [21].

We considered the following inclusion criteria: (1) age greater than 18 years old but less than 75 years, (2) PSVT was diagnosed by cardiac electrophysiological examination, (3) ineffective, poor ineffective, or intolerable drug treatment, (4) voluntary participation in this study and signed informed consent.

Exclusion criteria are as follows: (1) history of previous ablation treatment, (2) patients with severe cardiopulmonary and lung disease, liver, kidney insufficiency, and coagulation dysfunction who could not tolerate surgery, (3) history of myocardial infarction within 3 months, coronary artery bypass grafting or percutaneous coronary intervention, (4) history of stroke or transient ischemic attack (TIA) within 6 months, (5) patients implanted with artificial valves, pacemakers, cardiac defibrillators, (6) patients with New York Heart Association cardiac function grade III-IV [22], (7) imaging findings of thrombus in the left atrium appendage, (8) patients with second-degree type II or third-degree atrioventricular block (AVB).

Withdrawal criteria are as follows: (1) the patient voluntarily requested to withdraw from the study, (2) the patient developed life-threatening symptoms or illnesses, such as impaired consciousness and asphyxia.

### Sample size

We chose the SF-36 physiological function (PF) as the sample size calculation index based on 2 publications [23, 24] as well as the fact that the SF-36 consisted of 8 subscales and could not be used to calculate a total score. A total of 8 PSVT patients were recruited to complete the pre-trial at the Department of Cardiology, West China Hospital, Sichuan University, from October 2021 to March 2022. After receiving 2 different treatment strategies, the mean SF-36 PF score was 77.22 ± 7.12 in the PFA group and 70.27 ± 7.95 in the RFCA group, as shown in Supplementary file Schedule 1. Using a two-sided significance of 5% with an effect of 0.8, the sample size was 22 subjects per group. Assuming an attrition rate of 10%, the estimated sample size was 25 subjects per group, which should have been adequate to test our hypothesis.

### Randomization and blinding

Five physicians recruited subjects who met the inclusion criteria on an outpatient or inpatient unit, and within 2 days before surgery, the investigators used computer software to generate random numbers to divide patients into PFA and RFCA groups in a 1:1 ratio. Allocation concealment was the concealment of the random sequence number by the investigator assessing the outcome and the followers. Baseline data of PSVT patients were collected. Scale collectors, patients, and data analysts were unaware of the trial groupings, while surgeons were aware of the patient groupings.

### Intervention

Detailed baseline data of PSVT patients were collected after admission through cases. Echocardiography could determine the patient’s left atrial diameter and left ventricular ejection fraction. Preoperative transesophageal echocardiography (TEE) was useful to identify whether PSVT patients had left atrial thrombus, especially in the left atrial appendage. It is worth noting that if PSVT patients take oral anticoagulants, they should be discontinued at least 12 hours before surgery.

Patients were randomly assigned to the PFA and RFCA groups and were operated on by five attending physicians skilled in PSVT with more than 10 years of experience. The instruments used were all fully magnetically positioned 3D electrophysiological marker-mediated ablation systems developed by Sichuan Jinjiang Electronic Technology Company. All patients were followed up pre-procedure (T0), post-procedure (T1), and 3 months post-procedure (T2) by outpatient, telephone, or online.

### Outcome measurement

The primary outcome of this study was QOL, and the secondary outcome was psychological symptoms.

QOL was assessed using the 36-Item Short-Form Health Survey (SF-36), which was widely used to assess QOL [25]. The scale had 8 dimensions to evaluate health-related QOL, which were mainly divided into physical health and mental health, including physiological function (PF), role physical (RP), body pain (BP), general health (GH), vitality (VT), social function (SF), role emotional (RE), and mental health (MH). Higher scores indicated better QOL. We used the Chinese version of the SF-36, which has established reliability and validity in Chinese population [26]. Previously published data in arrhythmia samples showed that Cronbach’s *α* coefficient of 0.87 [27]. All patients were followed up by outpatient, telephone, or online communication at T0, T1, and T2 [28].

The psychological symptoms were mainly assessed by using the Hospital Anxiety and Depression Scale (HADS) [29]. The HADS was divided into an anxiety subscale (HADS-A) and a depression subscale (HADS-D), each with 7 items, and each item was scored using a 4-point scale from 0 to 3, with higher scores indicating higher anxiety or depression levels. The Chinese version of HADS has been widely validated [30, 31]. Cronbach’s *α* coefficient reported in a previous study on heart disease was ≥ 0.75 [32]. We evaluated the patients’ psychological symptoms at T0, T1, and T2.

### Data analysis

The demographic data, SF-36 scale, and HADS scale were entered into the SPSS 21.0 software (IBM Corp.) to build a database and validated by two researchers. Continuous variables that conformed to a normal distribution were expressed as mean ± standard deviation (SD), and continuous variables that did not conform to a normal distribution were expressed as median (interquartile range). Categorical variables were expressed as frequencies and percentages.

Wilcoxon and Fisher’s exact tests were used to assess differences in baseline characteristics between groups. An analysis of measurement covariance was implemented under a mixed model with change in the baseline as the dependent variable and the interaction of surgical modality, time, and surgical modality x time as the independent variables. Analysis of variance with univariate repeated measures data was performed if the information met spherical symmetry, and if it did not meet football symmetry, Bonferroni correction was used to adjust the *p*-value. The data were collated using the SPSS 21.0 software and plotted using the Prism8 software (GraphPad Prism, San Diego, CA).

## Results

### Baseline characteristics

**Fig. 1:**
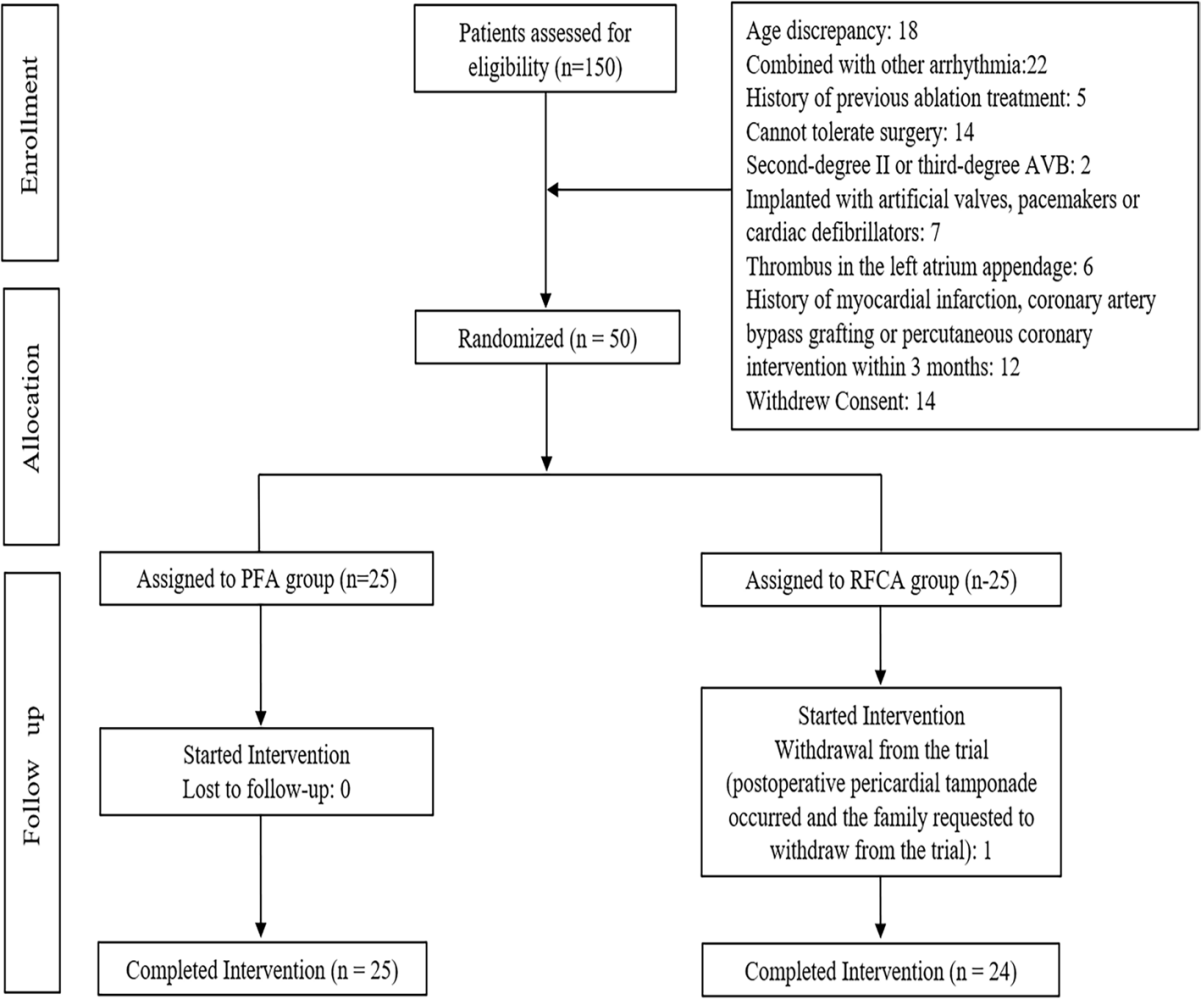
Flow chart

One hundred fifty patients were screened for this study. Eighteen were excluded because of age, 22 had other arrhythmias, 5 had a history of ablation, 14 had other serious medical conditions that could not tolerate the procedure, 2 had second-degree type II or third-degree AVB, 7 had implanted prosthetic valves, pacemakers, or defibrillators, 6 had thrombus in the left atrial or left atrial appendage, 12 had within 3 months history of myocardial infarction, coronary artery bypass graft, or percutaneous coronary intervention, and 14 declined to participate in this study. Fifty were recruited and randomly assigned to the trial. At 1 week post-procedure, 1 in the RFCA group requested to withdraw from the trial due to worsening clinical status. A total of 25 in the PFA group and 24 in the RFCA group were ultimately enrolled, as shown in Fig. 1.

There were no statistically significant differences (*P* > 0.05) between the two groups in the male ratio, age, weight, PSVT duration days, serum potassium at admission, hypertension, acute coronary artery disease (ACD), diabetes mellitus (DM), left atrial diameter (LAD) and left ventricular ejection fraction (LVEF), and PSVT type including 31 with dual atrioventricular node pathways (DAVNP), 13 with a left accessory pathway (AP), and 5 with right AP (see Table 1).

**Table 1:** Comparison of baseline clinical characteristics of the two groups

Variables	Overall (*n* = 49)	PFA (*n* = 25)	RFCA (*n* = 24)	*P* value
Age, mean (SD), years	50.29 (12.16)	50.52 (12.82)	50.04 (11.71)	0.464
Sex, *n* (%)				
Male	23 (47)	11 (44)	12 (50)	0.674
Female	26 (53)	14 (56)	12 (50)	
Weight, mean (SD), kg	65.02 (12.11)	65 (11.28)	65.04 (13.16)	0.292
Serum potassium, mean (SD), mmol/L	4.03 (0.54)	4.04 (0.60)	4.01 (0.49)	0.664
Duration of PSVT, mean (SD), d	1483.04 (1765.39)	1406.96 (1909.37)	1575.71 (1637.60)	0.204
PSVT type, *n* (%)				
DAVNP	31 (63)	16 (54)	15 (62)	0.866
Right AP	5 (10)	3 (12)	2 (8)	
Left AP	13 (26)	6 (24)	7 (29)	
Cardiac ultrasound indicators				
LAD, mean (SD), mm	32.90 (4.78)	32.56 (4.98)	33.25 (4.64)	0.559
LVEF, mean (SD), mm	67.35 (8.34)	66.84 (10.48)	67.88 (5.47)	0.139
Comorbidities, *n* (%)				
ACD	4 (8)	2 (8)	2 (8)	0.680
Hypertension	6 (12)	4 (16)	2 (8)	0.354
DM	4 (8)	2 (8)	2 (8)	0.680

*ACD* acute coronary disease; *AP* accessory pathway; *DAVNP* dual atrioventricular node pathways; *DM* diabetes mellitus; *LAD* left atrial diameter; *LVEF* left ventricular ejection fraction

### QOL

#### Between-group comparison

**Fig. 2:**
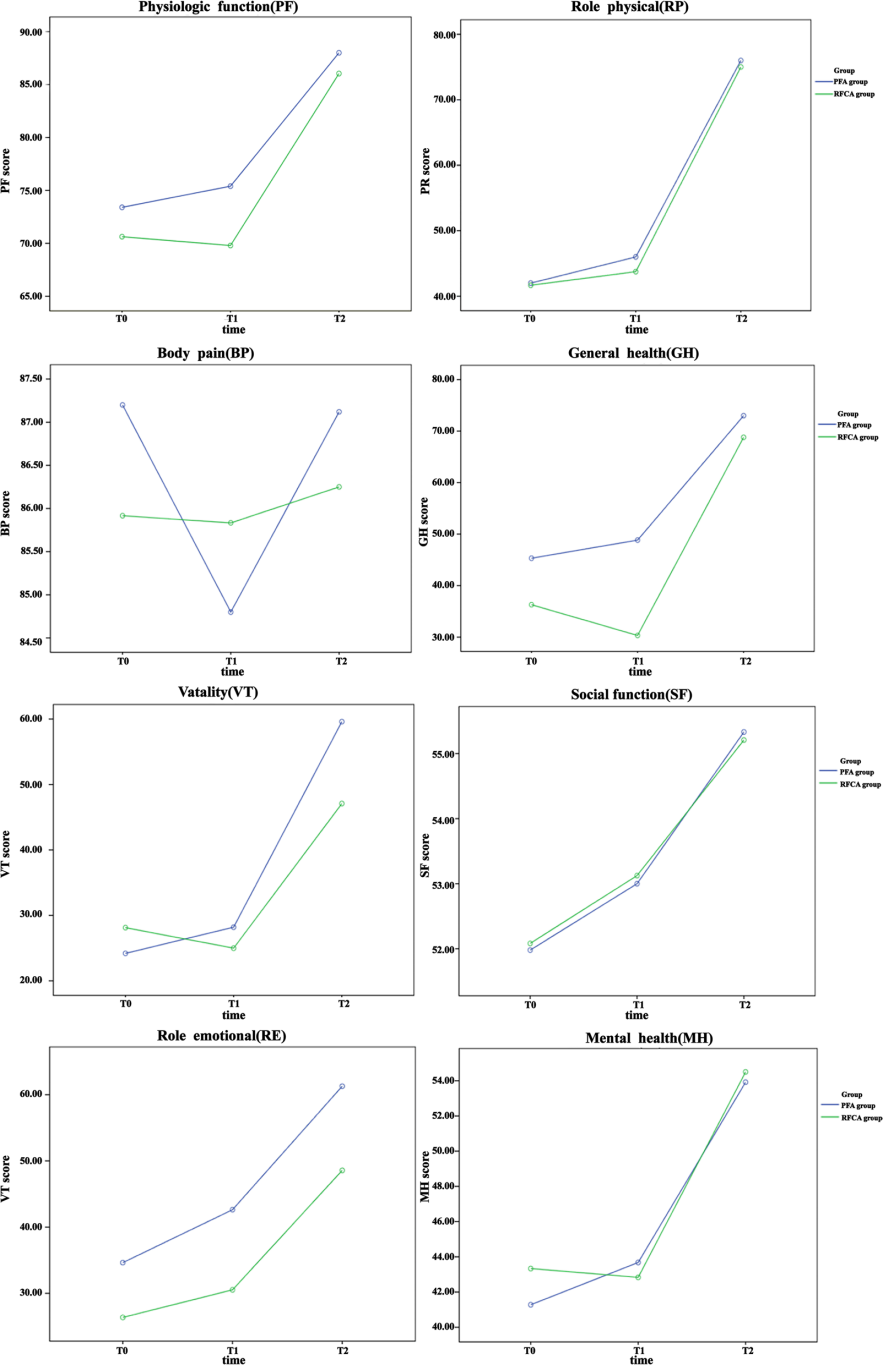
Primary outcome: Quality-of-Life Scores of 36-Item Short-Form Health Survey (SF-36). BP, body pain; GH, general health; MH, mental health; PF, physiological function; RE, role emotional; RP, role physical; T0, preoperative; SF, social function; T1, postoperative; T2, 3 months post-operative; VT, vitality. The left and right edges of the boxes indicate the interquartile range (IQR), the diamond inside indicates the mean value, and the line inside the box indicates the median value. The edges extend from each box to the furthest point within the ± 1.5 × IQR at the end of the box. Outliers are observations that are more extreme than the ± 1.5 × IQR and are indicated by small circles

There was a statistically significant difference in SF-36 PF scores between different surgical modalities (*F*= 7.76, *P* = 0.008). At T1, the SF-36 PF score improved significantly more in the PFA group than in the RFCA group (mean T0 score, 73.40 vs 70.6 [mean change 2.00 vs − 0.83]), with a mean treatment difference of 5.61 points (95% CI, 1.48–9.73, *P* = 0.009). There was a statistically significant difference in the SF-36 GH score between different surgical modalities (*F* = 14.40, *P* < 0.001). The SF-36 GH score was significantly higher in the PFA group than in the RFCA group at T1 (mean T0 score, 45.32 vs 36.29 [mean change 3.52 vs − 5.96]), with a mean treatment difference of 18.507 points (95% CI, 10.53–26.48, *P* < 0.001). In addition, there was no between-group difference in the remaining 6 subscale scores of the SF-36 (*P* > 0.05) (see Fig. 2 and Table 2).

**Table 2:** Between-group comparison: Quality-of-Life Scores of 36-Item Short-Form Health Survey (SF-36)

	Variables	PFA (*n* = 25)	RFCA (*n* = 24)	Between group *P* value
T0	Physical health, mean (SD)			
	PF	73.40 (1.22)	70.63 (1.25)	0.118
	RP	42.00 (6.31)	41.67 (6.44)	0.971
	BP	87.20 (2.31)	85.92 (2.36)	0.699
	GH	45.32 (3.27)	36.29 (3.34)	0.059
	VT	24.20 (2.82)	28.13 (2.88)	0.335
	Mental health, mean (SD)			
	SF	51.98 (2.74)	52.08 (2.80)	0.979
	RE	34.63 (4.65)	26.36 (4.75)	0.220
	MH	41.28 (1.88)	43.33 (1.92)	0.448
T1	Physical health, mean (SD)			
	PF	75.40 (1.44)	69.79 (1.47)	0.009
	RP	46.00 (5.92)	43.75 (6.04)	0.791
	BP	84.80 (2.32)	85.83 (2.36)	0.756
	GH	48.84 (2.78)	30.33 (2.83)	0.000
	VT	28.20 (2.42)	25.00 (2.47)	0.360
	Mental health, mean (SD)			
	SF	53.00 (3.07)	53.13 (3.14)	0.977
	RE	42.63 (5.04)	30.53 (5.14)	0.099
	MH	43.68 (1.99)	42.83 (2.03)	0.767
T2	Physical health, mean (SD)			
	PF	88.00 (0.74)	86.04 (0.76)	0.071
	RP	76.00 (5.88)	75.00 (5.99)	0.906
	BP	87.12 (2.37)	86.25 (2.42)	0.798
	GH	73.00 (2.13)	68.79 (2.17)	0.172
	VT	59.60 (4.64)	47.08 (4.74)	0.065
	Mental health, mean (SD)			
	SF	55.33 (3.24)	55.21 (3.31)	0.979
	RE	61.29 (5.94)	48.57 (6.06)	0.141
	MH	53.92 (2.58)	54.50 (2.63)	0.876

*BP* body pain; *GH* general health; *MH* mental health; *PF* physiological function; *RE* role emotional; *RP* role physical; *T0* preoperative; *SF* social function; *T1* postoperative; *T2* 3 months postoperative; *VT* vitality

#### Within-group comparison

T2 compared with T0 and T1, 6 SF-36 subscales were significantly improved by both ablation treatments (*P* < 0.05), but the PFA group scored generally higher than the RFCA group. Furthermore, the mean SF-36 BP score in the PFA group decreased by 0.08 points at T2 compared to T0 and increased by 2.32 points at T2 compared to T1, whereas in the RFCA group, it decreased by 0.33 points and 0.42 points at T2 compared to T1 and T0. The mean SF-36 SF score in the PFA group increased by 3.35 points and 2.33 points at T2 compared to T0 and T1. RFCA group increased by 3.13 points and 2.08 points. However, these changes were not statistically significant (*P* > 0.05) (see Fig. 2 and Table 3).

**Table 3:** Within-group comparison: Quality-of-Life Scores of 36-Item Short-Form Health Survey (SF-36)

	T0–T1		T0–T2		T1–T2	
*PFA (n = 25)*	*SMD*	*P* value	*SMD*	*P* value	*SMD*	*P* value
Physical health						
PF	− 2	0.274	− 14.60	0.000	− 12.60	0.000
RP	− 4	0.650	− 34	0.000	− 30.00	0.000
BP	2.40	0.520	0.08	1.000	− 2.32	0.188
GH	− 3.52	0.769	− 27.68	0.000	− 24.16	0.000
VT	− 4	0.057	− 35.40	0.000	− 31.40	0.000
Mental health						
SF	− 1.02	1.000	− 3.35	0.659	− 2.33	0.783
RE	− 7.99	0.053	− 26.66	0.000	− 18.66	0.000
MH	− 2.40	0.281	− 12.64	0.000	− 10.24	0.001
RFCA *(n = 24)*						
Physical health						
PF	0.83	1.000	− 15.42	0.000	− 16.25	0.000
RP	− 2.08	1.000	− 33.33	0.000	− 31.25	0.000
BP	0.08	1.000	− 0.33	1.000	− 0.42	1.000
GH	5.96	0.188	− 32.50	0.000	− 38.46	0.000
VT	3.13	0.208	− 18.96	0.000	− 22.08	0.000
Mental health						
SF	− 1.04	1.000	− 3.13	0.784	− 2.08	0.973
RE	− 4.16	0.647	− 22.21	0.000	− 18.05	0.000
MH	0.50	1.000	− 11.17	0.001	− 11.67	0.000

*SMD* standardized mean difference; *BP* body pain; *GH* general health; *MH* mental health; *PF* physiological function; *RE* role emotional; *RP* role physical; *T0* preoperative; *SF* social function; *T1* postoperative; *T2* 3 months post-operative; *VT*, vitality

### Psychological symptoms

#### Between-group comparison

**Fig. 3:**
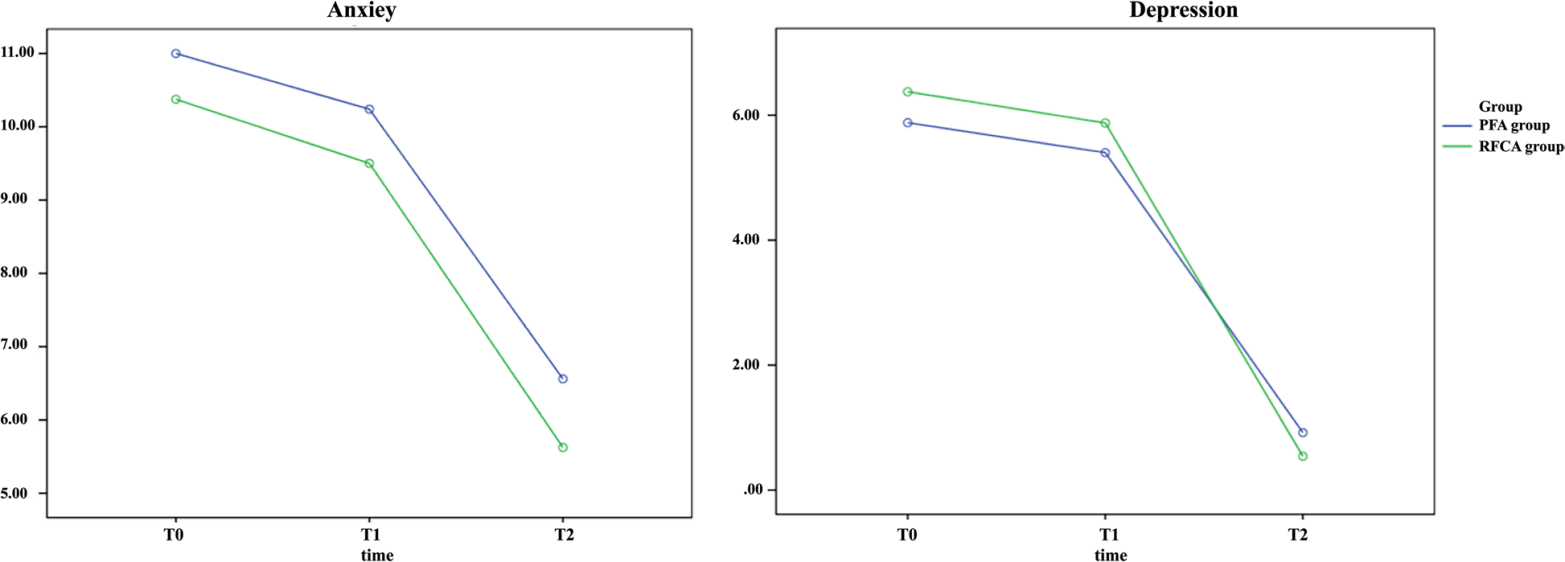
Secondary outcome: anxiety and depression scores on the Hospital Anxiety and Depression Scale (HADS). T0, preoperative; T1, postoperative; T2, 3 months post-operative. The left and right edges of the boxes indicate the interquartile range (IQR), the diamond inside indicates the mean value, and the line inside the box indicates the median value. The edges extend from each box to the furthest point within the ± 1.5 × IQR at the end of the box. Outliers are observations that are more extreme than the ± 1.5 × IQR and are indicated by small circles

Anxiety scores and depression scores on the HADS scale in the PFA and RFCA groups were not significantly different at T0, T1, and T2 (*P* > 0.05) (see Fig. 3 and Table 4).

**Table 4:** Between-group comparison: anxiety and depression scores on the Hospital Anxiety and Depression Scale (HADS)

	Variables	PFA (*n* = 25)	RFCA (*n* = 24)	*P* value
T0	Anxiety, mean (SD)	11 (0.55)	10.38 (0.56)	0.428
	Depression, mean (SD)	5.88 (0.46)	6.38 (0.47)	0.455
T1	Anxiety, mean (SD)	10.24 (0.58)	9.50 (0.59)	0.376
	Depression, mean (SD)	5.40 (0.53)	5.88 (0.54)	0.533
T2	Anxiety, mean (SD)	6.56 (0.36)	5.63 (0.37)	0.079
	Depression, mean (SD)	0.92 (0.17)	0.54 (0.17)	0.125

*T0* pre-procedure; *T1* post-procedure; *T2* 3 months post-procedure

#### Within-group comparison

In the PFA group T2 compared to T0, the mean anxiety score decreased by 4.44 points, the mean depression score decreased by 4.96 points, and the RFCA group decreased by 4.75 points and 5.83 points, and the difference was statistically significant (*P* < 0.05). In the PFA group T2 compared to T1, the mean anxiety score decreased by 3.68 points, the mean depression score decreased by 4.48 points, and the RFCA group decreased by 3.88 points and 5.33 points, and the difference was statistically significant (*P* < 0.05) (see Fig. 3 and Table 5).

**Table 5:** Within-group comparison: anxiety and depression scores on the Hospital Anxiety and Depression Scale (HADS)

	T0-T1		T0-T2		T1-T2	
	*SMD*	*P* value	*SMD*	*P* value	*SMD*	*P* value
*PFA (n = 25)*						
Anxiety	0.76	0.416	4.44	0.000	3.68	0.000
Depression	0.48	0.228	4.96	0.000	4.48	0.000
*RFCA (n = 24)*						
Anxiety	0.88	0.288	4.75	0.000	3.88	0.000
Depression	0.50	0.211	5.83	0.000	5.33	0.000

*SMD* standardized mean difference; *T0* preoperative; *T1* postoperative; *T2* 3 months postoperative

## Discussion

This was a single-center, randomized, single-blind, standard-controlled trial comparing QOL and psychological symptom outcomes in PSVT patients treated with RFCA and PFA. We found that PSVT patients treated with both ablation modalities showed significant improvement on the remaining 6 subscales of the SF-36 scale at T2, except BP and SF scores. Moreover, the improvement in the PF and GH subscales was significantly better at T1 in the PFA group compared to the RFCA group. Despite the HADS scores of the PFA and RFCA groups being statistically significant at T1 and T2 compared to T0, there were no significant differences between groups. Overall, our findings demonstrated that 2 surgical strategies improved PSVT patients’ QOL and psychological symptoms, while PFA performed better than RFCA in improving patients’ SF-36 PF and SF-36 GH.

In this study, we found that PSVT patients who underwent ablation improved on the SF-36 scale except for BP and SF scores, which were consistent with previous studies [33, 34]. Studies have shown that patients with arrhythmias have lower total QOL scores and lower scores on all dimensions than healthy people, with the main influencing factors related to symptom load, repeated visits, and medical costs [35, 36]. Long-term disease prevented patients from participating in social activities, but after ablation treatment, patients’ cardiac function improved, possibly reducing the distress caused by repeated hospitalizations and related symptoms caused by the disease [37, 38]. In contrast, the effect of 2 ablation procedures on SF-36 BP was not significant, which might be due to adverse experiences such as pain caused by puncture and surgical trauma [39]. Since PSVT patients included in this study usually had symptoms of sudden onset and sudden termination, and the duration and length of the attacks were inconsistent [40]. Therefore, the effect of pre- and post-ablation on patient SF-36 SF might not be significant, which was consistent with Bilanovic et al. [41].

After ablation, the mean SF-36 PH score of the PFA group was significantly higher than those of the RFCA group, and the difference was significant, indicating that PFA treatment was better able to improve the physiological functions of daily life of PSVT patients, such as walking and going up and down stairs. Successful ablation procedures could reduce or even completely eliminate the occurrence of supraventricular tachycardia, improved the patient’s somatic symptoms, and enhanced the body’s function [42], whereas different ablation procedures led to differences in SF-36 PH scores that might be related to the ablation mechanism, timing, and postoperative pain [43]. RFCA has thermal properties, and the damage caused by RFCA was significantly different from that of PFA. RFCA treatment possibly resulted in the formation of scar tissue, endothelial hyperplasia, and even necrotic myocardium [44], whereas PFA treatment produced tissue damage mainly in the form of uniform fibrosis without endocardial rupture [45, 46]. It is worth mentioning that RFCA energy could be delivered at higher power in a shorter period, so the heating process might be shorter but still take more time [47]. In contrast, the energy supply of PFA was almost instantaneous, allowing the required energy level to be achieved in a very short time, reducing the difficulty of the procedure. This might shorten the duration of the procedure and less pain, thereby increasing exercise tolerance and improving the patient’s physical function [48].

The postoperative SF-36 GH score of the PFA group was higher than that of the RFCA group, suggesting that PFA procedure could effectively improve the patients’ overall evaluation of their self-physical health status. This might be due to the fact that PFA was different from the RFCA procedure based on thermal effects. PFA was able to selectively ablate abnormal cardiac discharge tissue while preserving blood vessels, nerves, and normal tissues [48], thereby reducing postoperative discomfort, minimizing complications, and improving the patient experience. This was similar to the results of a randomized clinical trial study [23]. As the ablation time prolongs, the impact of 2 procedures on QOL decreased, and the difference was not significant. This might be due to the fact that the conditions and discomforts caused by different procedures were controlled, thus ensuring the patient’s basic daily life needs.

Our results found that two different ablation procedures could positively affect anxiety and depression in PSVT patients. This might be related to the patients’ improved psychological status due to the relief of their symptoms and improved QOL after undergoing the ablation procedure. These results were consistent with those obtained in previous studies of patients with atrial fibrillation undergoing ablation therapy [49–51].

This might be due to a variety of reasons, such as better treatment, more social support, and a normal return to life, where the patient’s anxiety and depression improve best [52]. There was no significant difference in anxiety and depression scores between the RFCA group and the PFA group at T1 and T2 (*P* > 0.05). This might be because both surgical approaches could improve the patient’s symptoms as a way to promote recovery from the psychological state. Thus, our results suggested that the psychological benefits of ablation observed in other arrhythmia populations were also applicable to patients with PSVT.

There were also some limitations in this study. First, none of the surgeon arrangements were randomized, but all surgeons had considerable experience and were skilled in both RFCA and PFA. Moreover, each ablation procedure was performed by fixed surgeons, and all surgeons were not involved in data collection. Therefore, we believed that this bias had little influence on the data. Second, for PSVT patients after undergoing ablation, only short-term follow-up results of postoperative patients were currently available. To better understand QOL, anxiety, and depression of PSVT patients after different ablation procedures, further long-term follow-up studies of ablation patients are needed. Third, the sample size of this study was small, and the representativeness and generalizability of the results might be limited. In the future, the sample size of the study could be further expanded, while multicenter, large-sample randomized controlled trials could be conducted to provide more scientific and effective evidence for the further application of PFA. Finally, our outcome measures were the SF-36 and HADS scales, which might have produced a Hawthorne effect [53]. In our study, PSVT patients were unaware of their subgroups, which could minimize the possibility of subjects changing their behavior and better reflect the real situation.

## Conclusion

Our findings demonstrated that 2 surgical strategies improved PSVT patients’ QOL and psychological symptoms, while PFA performed better than RFCA in improving patients’ SF-36 PF and SF-36 GH. Given the inevitable potential damage to normal tissue, long ablation times, and poor patient experience associated with RFCA ablation, the PFA technique took into account the patient experience while achieving a better treatment outcome. Our findings possibly provided a reference for the application of PFA in adults with PSVT and facilitated the development of new technologies.

### Supplementary Information

**Additional file 1.** Supplementary Material 1.

**Additional file 2.** Supplementary Material 2.

## Data Availability

All original data and materials appearing in the study can be further consulted by contacting the corresponding author.

## Abbreviations

PFA: Pulse field ablation
RFCA: Radiofrequency catheter ablation
QOL: Quality of life
PSVT: Paroxysmal supraventricular tachycardia
SF-36: 36-Item Short-Form Health Survey
HADS: Hospital Anxiety and Depression Scale
TEE: Transesophageal echocardiography
ACD: Artery disease
DM: Diabetes mellitus
LVEF: Left ventricular ejection fraction
DAVNP: Dual atrioventricular node pathways
