# Supplementary material for: Comparing the effects of pulsed and radiofrequency catheter ablation on quality of life, anxiety, and depression of patients with paroxysmal supraventricular tachycardia: a single-center, randomized, single-blind, standard-controlled trial
Source: Trials. 2024 Feb 24;25:146. doi: 10.1186/s13063-024-07971-8 (PMC10893749; doi:10.1186/s13063-024-07971-8)
Supplement: Supplementary file 1 — Additional file 1. Supplementary Material 1. [file 13063_2024_7971_MOESM1_ESM.docx]

**Schedule 1.** Pre-trial: Quality-of-Life Scores of 36-Item Short-Form Health Survey (SF-36).

BP, body pain; GH, general health; MH, mental health; PF, physiological function; RE, role

| Variables | T0 | | T1 | | T2 | |
| --- | --- | --- | --- | --- | --- | --- |
|  | PFA  （n = 4） | RFCA  （n = 4） | PFA  （n = 4） | RFCA  （n = 4） | PFA  （n = 4） | RFCA  （n = 4） |
| PF | 72.50(6.45) | 71.25(6.29) | 77.00(4.76) | 70.00(7.07) | 88.75(4.79) | 83.75(2.50) |
| RP | 37.50(25.00) | 25.00(28.87) | 37.50(25.00) | 25.00(28.87) | 75.00(28.87) | 62.50(25.00) |
| BP | 86.50(17.99) | 89.50(12.79) | 86.50(17.99) | 89.50(12.79) | 86.50(17.99) | 89.50(12.79) |
| GH | 33.00(12.88) | 26.25(4.79) | 39.75(14.29) | 22.50(5.00) | 63.75(13.87) | 68.75(11.64) |
| VT | 23.75(11.09) | 32.50(15.00) | 23.75(11.09) | 32.50(15.00) | 55.00(17.32) | 60.00(20.00) |
| SF | 50.00(17.68) | 50.00(10.21) | 50.00(17.68) | 53.12(11.97) | 50.00(17.68) | 59.38(11.97) |
| RE | 24.98(16.65) | 16.65(19.23) | 41.63(31.88) | 24.98(16.65) | 66.63(27.23) | 41.63(16.65) |
| MH | 42.00(4.00) | 46.00(15.14) | 42.00(4.00) | 46.00(15.14) | 62.00(12.00) | 55.00(15.09) |

emotional; RP, role physical; T0, preoperative; SF, social function; T0, pre-procedure; T1, post-procedure; T2, 3 months post-procedure; VT, vitality.
